# Pathogen distribution and liver injury severity in children with community-acquired pneumonia complicated by liver injury in Suzhou, China

**DOI:** 10.3389/fped.2025.1665002

**Published:** 2025-09-15

**Authors:** Kun Wang, ZhengJiayi Li, Fangfang Cheng

**Affiliations:** ^1^ Department of Infectious Diseases, Children’s Hospital of Soochow University, Suzhou, China; ^2^ Department of Pediatrics, Qingdao Hospital, University of Health and Rehabilitation Sciences, Qingdao, China

**Keywords:** children, community-acquired pneumonia, liver injury, pathogen distribution, severity

## Abstract

**Background:**

Liver injury is a extrapulmonary complication of community-acquired pneumonia (CAP). However, limited data exist on the pathogen distribution and severity of liver injury in children with CAP-associated liver injury. This study aimed to investigate the characteristics of pathogen distribution and the severity of liver injury in children with CAP complicated by liver injury in the Suzhou area.

**Methods:**

A retrospective study was conducted on children with CAP hospitalized at the Children's Hospital of Soochow University between January 2018 and December 2022. The study included children aged over 28 days to under 18 years, categorized into the following age groups: >28 days to 1 year, >1–3 years, >3–5 years, and >5 years. Laboratory examination results, pathogens, and characteristics of liver injury were analyzed.

**Results:**

Among the 1525 children with CAP complicated by liver injury, the male-to-female ratio was 1.4:1. Mild elevation of transaminases were observed in 1,403 cases. In the same age group, there were differences in the proportion of cases with varying degrees of liver injury (*p* *<* 0.05). Among the four age groups, both the number of cases and the incidence of liver injury were highest in the >28 days to 1-year-old group. The incidence of liver injury was higher in children with severe community-acquired pneumonia (SCAP). Additionally, the distribution of pathogens varied significantly among age groups (*p* < 0.001). Children with severe liver injury were mostly accompanied by *Mycoplasma pneumoniae (M. pneumoniae)* infection (88.89%). Alanine aminotransferase (ALT) levels also varied significantly based on age group, pathogen type, and pneumonia severity (*p* < 0.05). Importantly, none of the children progressed to liver failure.

**Conclusions:**

In the Suzhou area, children with CAP aged >28 days to 1 year were the most susceptible to liver injury, with mild elevation of transaminases being the most common presentation. Special attention was required for children with CAP complicated by *M. pneumoniae* infection, as they carried a higher risk of severe liver injury. Children with SCAP were more prone to liver injury. Additionally, pathogen distribution varied across different age groups in children with CAP complicated by liver injury.

## Background

CAP is one of the most prevalent childhood diseases, with approximately 120 million new cases reported annually. It remains the leading cause of death in children under 5 years old, posing a significant threat to pediatric health (1, 2). CAP can be caused by various pathogens, including *viruses*, *bacteria*, and *M. pneumoniae* (3). The most common symptoms include fever, and cough. However, some children may also exhibit extrapulmonary manifestations in other systems, with liver injury as a well-recognized complication (4).

As one of the most important organs in the human body, the liver performs vital physiological functions in aspects such as synthesis, metabolism, detoxification, and immunity. There are many causes of liver injury in children, including infections, drugs, poisoning, autoimmune diseases, and inherited metabolic diseases. Clinically, symptoms such as anorexia, vomiting, and abdominal distension may appear, or they may be accompanied by signs such as jaundice of the skin and sclera, and hepatomegaly (5). Infection is the most common cause of liver injury, and the severity and clinical characteristics of liver injury caused by infections with different pathogens vary. Studies have found that after *COVID-19* infection, the incidence and severity of liver injury differ among patients of different ages. Neonates have the mildest liver injury, and the incidence of liver injury in children is much lower than that in adults (6).

At present, the pathogenesis of CAP complicated by liver injury has not been fully clarified. The possible mechanisms are considered as follows. First, CAP-associated liver injury may be directly caused by pathogen infection. *Viral* infection of hepatocytes and bile duct epithelial cells is the cause of liver injury. Pathology of liver tissue shows hepatocyte degeneration, focal necrosis with neutrophil infiltration, and congestion of hepatic sinusoids (7). Secondly, liver tissue contains a large number of cells involved in immune responses and possesses important immune defense and immune regulation functions. Pathogen infection can trigger a systemic inflammatory response. The increased secretion of inflammatory factors such as interleukin-1, interleukin-6, and tumor necrosis factor-α leads to a cytokine storm, which further results in immune-mediated liver injury (8). In addition, children with pneumonia are often accompanied by wheezing and shortness of breath, and may have hypoxemia. The liver has a high demand for oxygen and is easily affected by hypoxia. A reduction in oxygen supply and lipid accumulation in hepatocytes can lead to hepatocyte death (9).

Currently, limited research exists on the severity of liver injury in children with CAP and the distribution of causative pathogens, with no relevant studies reported in Suzhou. This study examines the etiological findings and liver injury severity of 1,525 children with CAP complicated by liver injury who were hospitalized at the Children's Hospital of Soochow University between January 2018 and December 2022. The study aims to provide clinicians with a clearer understanding of the severity of liver injury in children with CAP and the pathogen distribution patterns, facilitating improved diagnosis and management of affected patients.

## Materials and methods

### Study subjects

**Figure 1:**
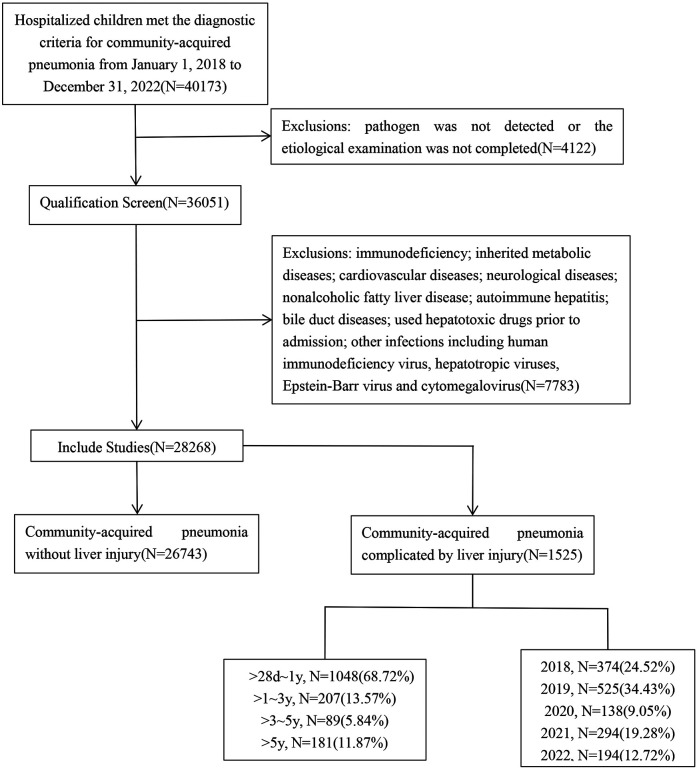
Flowchart of this study. A retrospective study was conducted on children with community-acquired pneumonia (CAP) hospitalized at the Children's Hospital of Soochow University between January 2018 and December 2022, with a total of 40,173 CAP children. According to the inclusion and exclusion criteria, a total of 28,268 children with CAP were enrolled. Based on the presence or absence of liver injury, these 28,268 CAP children were divided into two groups: CAP without liver injury group and CAP complicated by liver injury group.

This retrospective study was conducted on children with CAP who received treatment at the Children's Hospital of Soochow University from January 2018 to December 2022. Children were included in the study upon meeting the following criteria: age between >28 days and <18 years; fulfilled the diagnostic criteria for CAP; pathogens were identified in the CAP children; and complete clinical data were available. Children with immunodeficiency, inherited metabolic diseases, other infections including *human immunodeficiency virus*, *hepatotropic viruses*, *Epstein–Barr virus*, and *cytomegalovirus* were excluded. Based on the presence or absence of liver injury, these CAP children were divided into two groups: CAP without liver injury group and CAP complicated by liver injury group (Figure 1). The study received ethical approval from the Ethics Committee of the Children's Hospital of Soochow University, China (approval number: 2023CS171).

### Diagnostic criteria

#### Diagnostic criteria for community-acquired pneumonia

The patient presents with respiratory symptoms. Fever, cough, and wheezing are the most common symptoms of CAP. Fixed moist rales can be heard on lung auscultation, and chest imaging suggests pneumonia. Etiological detection can identify the infectious pathogen (10).

#### Diagnostic criteria for severe pneumonia

Children with CAP are diagnosed with severe pneumonia if they present with one or more of the following conditions: (1) Poor general physical condition; (2) Consciousness disorder; (3) Oxygen saturation <92%, cyanosis or tachypnea, with significantly increased respiratory rate (infants >70 breaths/min, children over 1 year old >50 breaths/min); (4) Ultra-high fever (temperature >41℃) or persistent moderate-high fever for more than 5 days (temperature 38℃–41℃); (5) With dehydration or symptoms of anorexia; (6) Chest radiograph or chest CT shows ≥2/3 infiltrate in one lung, multilobar infiltration, pleural effusion, pneumothorax, atelectasis, pulmonary necrosis, or lung abscess; (7) Occurrence of extrapulmonary complications such as septic shock, and acute renal failure (10).

#### Diagnostic criteria for liver injury and the degree of severity

Liver injury was defined as an elevation in liver enzyme levels of at least twice the upper limit of normal. An ALT level twice the upper limit of normal and ≤199 U/L indicates mild injury; 200–600 U/L indicates moderate injury; and >600 U/L indicates severe injury (11).

### Data collection

The demographic and laboratory characteristics were collected. Demographic characteristics included age, gender. The laboratory values included respiratory etiological detection, MP antibody and liver function tests.

### Etiological detection

Nasopharyngeal aspirates collected after admission were analyzed for the presence of *respiratory syncytial virus (RSV)*, *human parainfluenza virus (HPIV)*, and *adenovirus (ADV)* using direct immunofluorescence. Reverse transcriptase polymerase chain reaction (RT-PCR) was performed to detect *influenza A virus (FluA)*, *influenza B virus (FluB)*, *M. pneumoniae*, *human bocavirus (hBoV)*, *human metapneumovirus (hMPV)*, and *human rhinovirus (HRV)*. *Bacterial* detection in nasopharyngeal aspirates was conducted via culture-based methods. Samples were cultured on blood agar plates following clinical standard culture procedures, and bacterial species were identified by quantitative analysis.

### Statistical analysis

Statistical analyses were conducted using SPSS 25.0 software (IBM, Armonk, NY, USA). Data were presented as the number [*n* (%)], with categorical data analyzed using the chi-square, continuity correction or Fisher's exact test, as appropriate. Non-normally distributed data were expressed as medians (interquartile ranges). Comparisons of quantitative variables across different groups were performed using the Mann–Whitney *U*-test and Kruskal–Wallis *H*-test. A *p*-value <0.05 was considered statistically significant.

## Results

### Children characteristics

A total of 28,268 children with CAP were included in the study. In these CAP children, 1,525 had extrapulmonary complications with liver injury. Among them, 896 were males (58.75%), and 629 were females (41.25%), resulting in a male-to-female ratio of 1.4:1. The youngest child was 1 month old, while the oldest was 204 months old. The median age was 6 months (interquartile range: 3–12 months). Age distribution analysis showed that 1,048 children (68.72%) were aged >28 days to 1 year, 207 (13.57%) were aged >1–3 years, 89 (5.84%) were aged >3–5 years, and 181 (11.87%) were aged >5 years (Figure 1).

### Pathogenic characteristics in children with CAP complicated by liver injury across age groups

**Figure 2:**
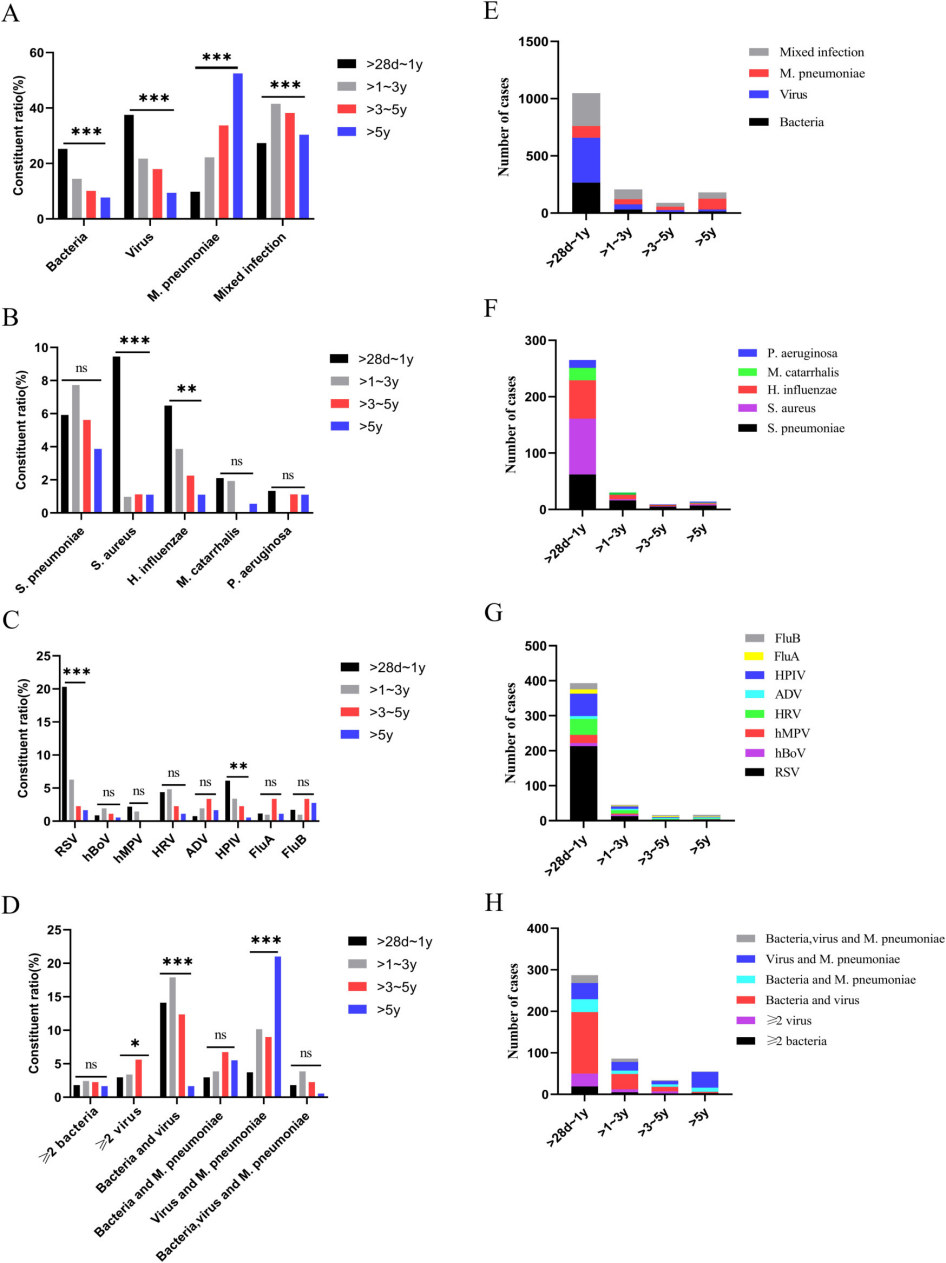
Pathogen detection in children with community-acquired pneumonia complicated by liver injury. **(A–D)** Constituent ratio of pathogens, **(E–H)** Number of pathogens. *p < 0.05, **p < 0.01, ***p < 0.001, ns, no significant difference. *S. pneumoniae*, *Streptococcus pneumoniae*; *S. aureus*, *Staphylococcus aureus*; *H. influenzae*, *Haemophilus influenzae*; *M. catarrhalis*, *Moraxella catarrhalis*; *P. aeruginosa*, *Pseudomonas aeruginosa*; RSV, respiratory syncytial virus; hBoV, human bocavirus; hMPV, human metapneumovirus; HRV, human rhinovirus; ADV, adenovirus; HPIV, human parainfluenza virus; FluA, influenza A virus; FluB, influenza B virus; *M. pneumoniae*, *Mycoplasma pneumoniae*.

The >28 days to 1-year age group had the highest number of cases, while the >3–5-year age group had the least. In the >28 days to 1-year group, *viral* infections were the most prevalent, and *M. pneumoniae* infections were the least common. As age increased, the proportions of *viral* and *bacterial* infections gradually declined, while the proportion of *M. pneumoniae* infections increased. There were differences in pathogen distribution among different age groups (*p* < 0.001) (Table 1). Within each age group, the predominant bacterial pathogen varied. In the >28 days to 1-year group, *Staphylococcus aureus (S. aureus)* was the most frequently detected pathogen. In the other three age groups, *Streptococcus pneumoniae (S. pneumoniae)* was the most frequently detected pathogen. *RSV* was the most prevalent viral infection in the >28 days to 1-year and >1–3-year groups. *ADV*, *FluA*, and *FluB* had the highest detection rates in the >3–5-year group, while *FluB* was the most frequently detected in the >5-year group. The constituent ratio of the number of cases of *S. aureus*, *Hemophilus influenzae (H. influenzae)*, *RSV*, *HPIV*, and *M. pneumoniae* varied significantly across the different age groups (*p* < 0.05) for the four CAP age groups with liver injury (Figure 2).

**Table 1:** Pathogen detection in children with community-acquired pneumonia complicated by liver injury across various age groups.

Age	>28 days–1 years (*n* = 1,048)	>1–3 years (*n* = 207)	>3–5 years (*n* = 89)	>5 years (*n* = 181)
Bacteria (*n* = 318)	265 (25.29)	30 (14.49)	9 (10.11)	14 (7.72)
Virus (*n* = 471)	393 (37.50)	45 (21.74)	16 (17.98)	17 (9.40)
*M. pneumoniae* (*n* = 274)	103 (9.83)	46 (22.22)	30 (33.71)	95 (52.49)
Mixed infection (*n* = 462)	287 (27.38)	86 (41.55)	34 (38.20)	55 (30.39)
*χ*^2^ value	211.946	23.554	25.311	183.868
*p-*value	<0.001	<0.001	<0.001	<0.001

The data presented as *n* (%). *M. pneumoniae*, *Mycoplasma pneumoniae*.

### Characteristics of mixed pathogen infections

Children with CAP complicated by liver injury in each age group exhibited mixed infections, with a total of 462 cases (30.30%). The highest proportion of mixed infections was observed in children aged >1–3 years (Table 1). The distribution of mixed infections varied significantly among different age groups (*p* < 0.001) (Figure 2). There were differences in the distribution of different types of mixed infections in the age groups of >28 days to 1 year and >5 years (*p* < 0.001) (Table 2). In the >28 days to 1-year, >1–3-year, and >3–5-year groups, *bacteria*-*virus* mixed infections were the most common. However, in the >5-year group, *virus*-*M. pneumoniae* mixed infections were the most prevalent (Table 2). Among children with *bacteria*-*virus* mixed infections in the >28 days to 1-year group, the most frequently observed combinations included *RSV* mixed with *S. aureus* or *S. pneumoniae*, or *H. influenzae*. Among children with *virus*-*M. pneumoniae* mixed infections in the >5-year group, the most common combinations involved *M. pneumoniae* mixed with *HRV* or *HPIV*.

**Table 2:** Mixed infections in children with community-acquired pneumonia complicated by liver injury across various age groups.

Age	>28 days–1 years (*n* = 287)	>1–3 years (*n* = 86)	>3–5 years (*n* = 34)	>5 years (*n* = 55)
≥2 bacteria (*n* = 29)	19 (6.62)	5 (5.81)	2 (5.88)	3 (5.45)
≥2 virus (*n* = 43)	31 (10.80)	7 (8.14)	5 (14.71)	0 (0.00)
Bacteria and virus (*n* = 199)	148 (51.57)	37 (43.03)	11 (32.35)	3 (5.45)
Bacteria and *M. pneumoniae* (*n* = 55)	31 (10.80)	8 (9.30)	6 (17.65)	10 (18.19)
Virus and *M. pneumoniae* (*n* = 106)	39 (13.59)	21 (24.42)	8 (23.53)	38 (69.09)
Bacteria, virus and *M. pneumoniae* (*n* = 30)	19 (6.62)	8 (9.30)	2 (5.88)	1 (1.82)
χ^2^ value	44.325	2.176	3.181	82.431
*p-*value	<0.001	0.824	0.672	<0.001^a^

The data presented as *n* (%). *M. pneumoniae*, *Mycoplasma pneumoniae*.

^a^ Fisher's exact test.

### Pathogen distribution characteristics in children with CAP and those with liver injury

**Figure 3:**
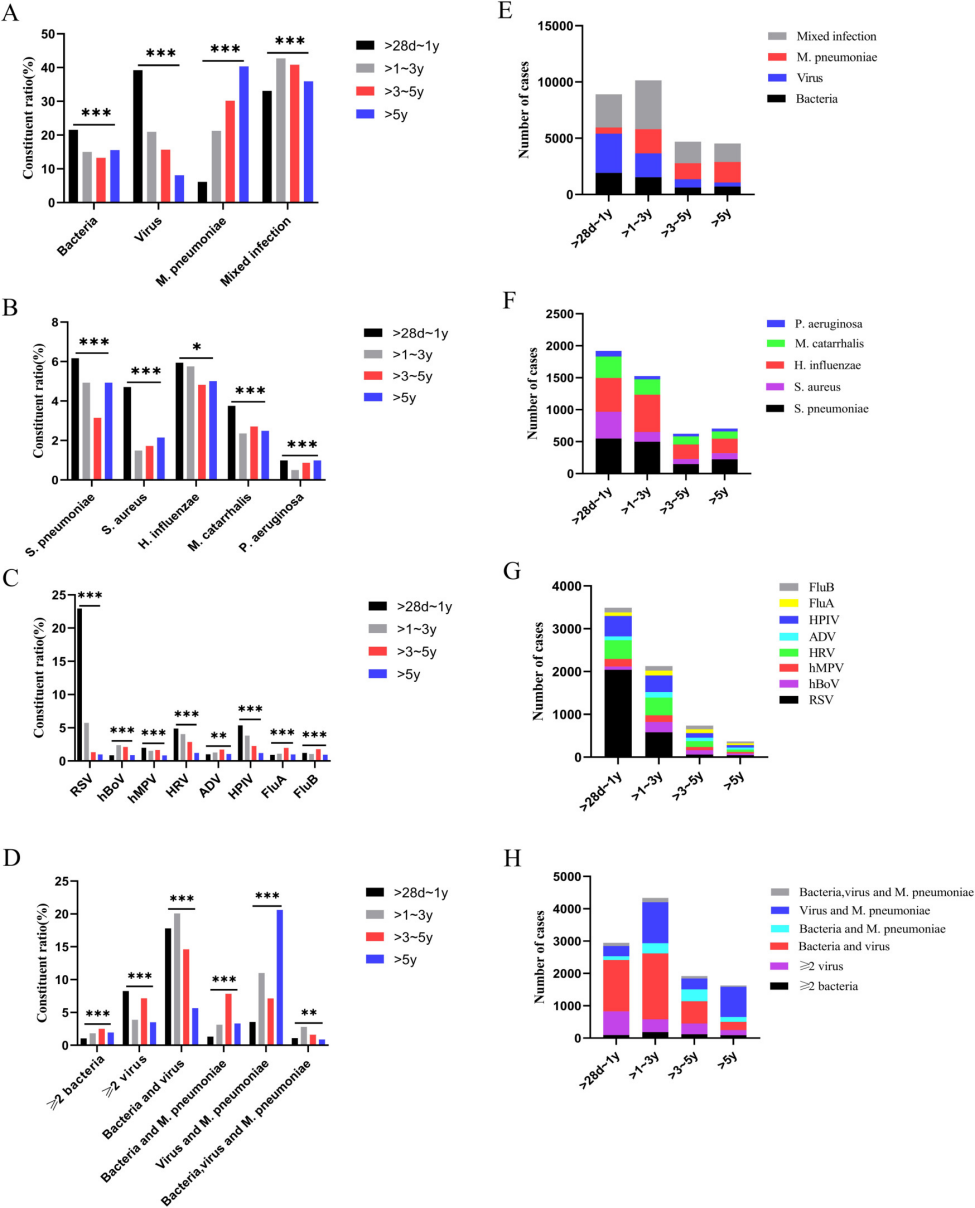
Pathogen detection in children with community-acquired pneumonia. **(A–D)** Constituent ratio of pathogens, **(E–H)** Number of pathogens. *p < 0.05, **p < 0.01***, *p* < 0.001. *S. pneumoniae*, *Streptococcus pneumoniae*; *S. aureus*, *Staphylococcus aureus*; *H. influenzae*, *Haemophilus influenzae*; *M. catarrhalis*, *Moraxella catarrhalis*; *P. aeruginosa*, *Pseudomonas aeruginosa*; RSV, respiratory syncytial virus; hBoV, human bocavirus; hMPV, human metapneumovirus; HRV, human rhinovirus; ADV, adenovirus; HPIV, human parainfluenza virus; FluA, influenza A virus; FluB, influenza B virus; *M. pneumoniae*, *Mycoplasma pneumoniae*.

**Figure 4:**
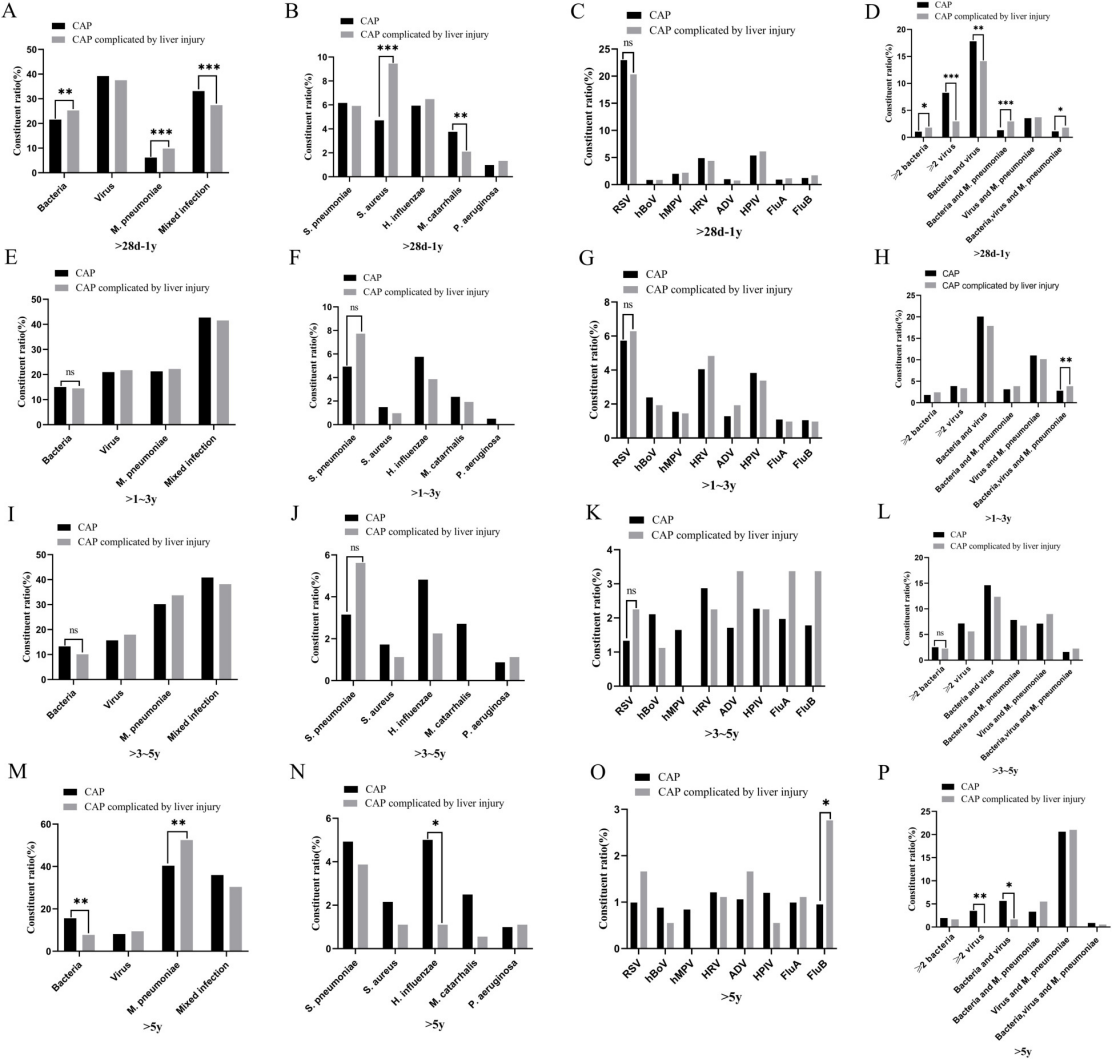
Distribution of pathogens in children with community-acquired pneumonia and those with liver injury. **(A–D)** >28 days–1-year group, **(E–H)** >1–3-year group, **(I–L)** >3–5-year group, **(M–P)** >5-year group. *p < 0.05, **p < 0.01***, *p* < 0.001, ns, no significant difference. *S. pneumoniae*, *Streptococcus pneumoniae*; *S. aureus*, *Staphylococcus aureus*; *H. influenzae*, *Haemophilus influenzae*; *M. catarrhalis*, *Moraxella catarrhalis*; *P. aeruginosa*, *Pseudomonas aeruginosa*; RSV, respiratory syncytial virus; hBoV, human bocavirus; hMPV, human metapneumovirus; HRV, human rhinovirus; ADV, adenovirus; HPIV, human parainfluenza virus; FluA, influenza A virus; FluB, influenza B virus; *M. pneumoniae*, *Mycoplasma pneumoniae*.

There were differences in the constituent ratios of pathogen infections among children with CAP across different age groups (*p* < 0.05) (Figure 3). The pathogen distribution varied between children with CAP and those with CAP complicated by liver injury. In the group aged >28 days to 1 year, there were differences in the constituent ratios of *bacteria*, *M. pneumoniae*, mixed infections, *S. aureus*, *Moraxella catarrhalis (M. catarrhalis)*, ≥2 *bacterial* infections, ≥2 *viral* infections, as well as *bacteria*-*virus*, *bacteria*-*M. pneumoniae*, and *bacteria*-*virus*-*M. pneumoniae* mixed infections between children with CAP and those with CAP complicated by liver injury. In the >1–3-year group, there were differences in *bacteria*-*virus*-*M. pneumoniae* mixed infections between the children with CAP and those with CAP complicated by liver injury. In the >5-year group, there were differences in the constituent ratios of *bacteria*, *M. pneumoniae*, *H. influenzae*, *FluB*, ≥2 *viruses*, and *bacteria*-*virus* mixed infections between the children with CAP and those with CAP complicated by liver injury (*p* < 0.05). For other pathogen infections, no differences were observed between children with CAP and those with CAP complicated by liver injury in any age group (Figure 4).

### Liver injury incidence in children with CAP

**Figure 5:**
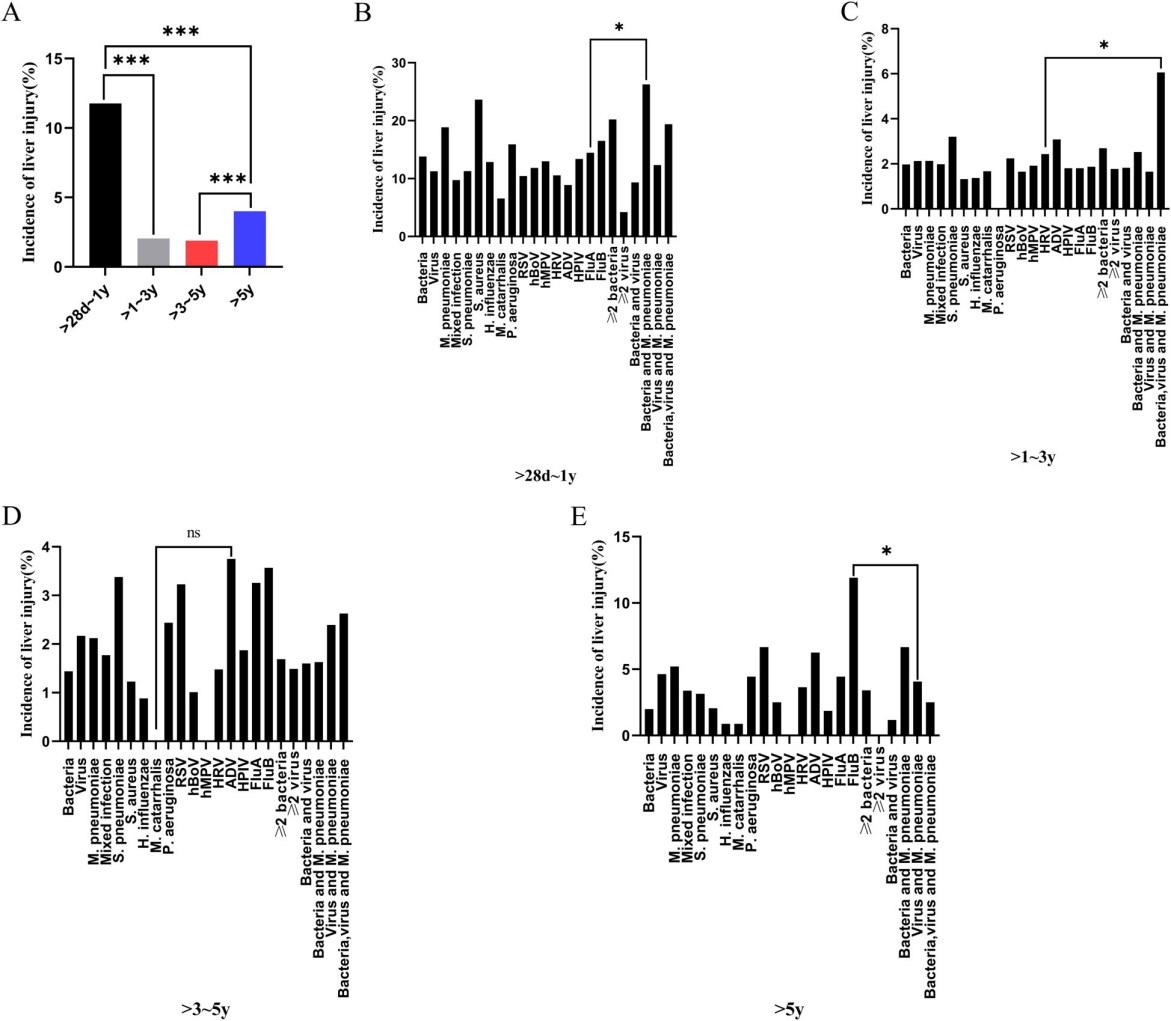
Incidence of liver injury in children with community-acquired pneumonia across different age groups and pathogen types. **(A)** Incidence of liver injury in children with community-acquired pneumonia across different age groups ***p < 0.001; **(B)** Incidence of liver injury in community-acquired pneumonia caused by different pathogens in the >28 days to 1-year group *p < 0.05; **(C)** Incidence of liver injury in community-acquired pneumonia caused by different pathogens in the >1–3-year group *p < 0.05; **(D)** Incidence of liver injury in community-acquired pneumonia caused by different pathogens in the >3–5-year group, ns, no significant difference; **(E)** Incidence of liver injury in community-acquired pneumonia caused by different pathogens in the >5-year group *p < 0.05. *S. pneumoniae*, *Streptococcus pneumoniae*; *S. aureus*, *Staphylococcus aureus*; *H. influenzae*, *Haemophilus influenzae*; *M. catarrhalis*, *Moraxella catarrhalis*; *P. aeruginosa*, *Pseudomonas aeruginosa*; RSV, respiratory syncytial virus; hBoV, human bocavirus; hMPV, human metapneumovirus; HRV, human rhinovirus; ADV, adenovirus; HPIV, human parainfluenza virus; FluA, influenza A virus; FluB, influenza B virus; *M. pneumoniae*, *Mycoplasma pneumoniae*.

The incidence of liver injury was 11.77% in the >28 days to 1-year age group, which was significantly higher than that in the other three age groups (*p* < 0.001) (Figure 5). In this group (>28 days to 1 year), the highest incidence of liver injury (26.27%) was observed in children with mixed *bacterial*, and *M. pneumoniae* infection. In the >1–3-year group, the highest incidence of liver injury (6.06%) occurred in children with mixed *bacterial*, *viral*, and *M. pneumoniae* infections. In the >5-year group, children with *FluB* had the highest incidence of liver injury (11.9%) (*p* < 0.05) (Figure 5).

### Liver function in children with CAP complicated by liver injury

**Figure 6:**
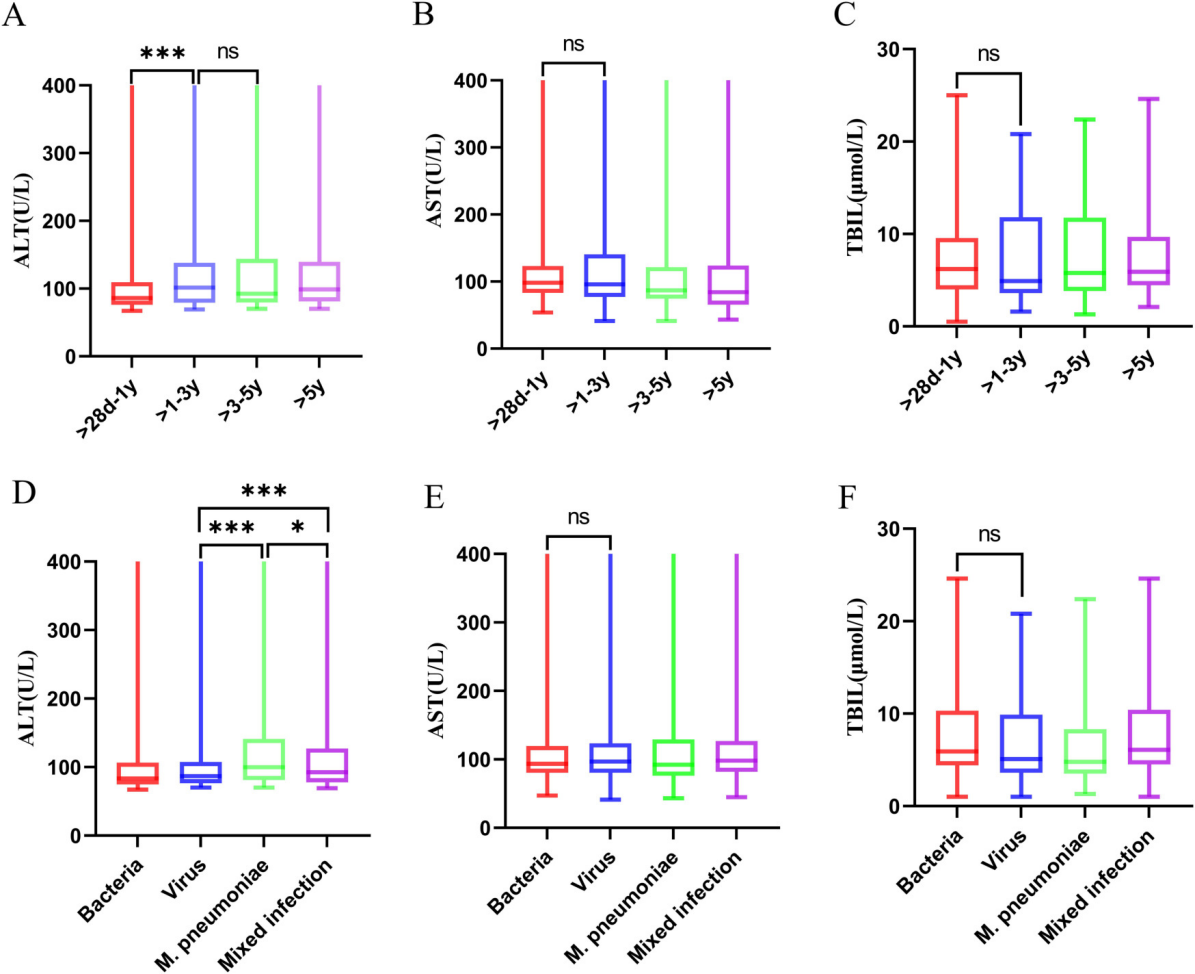
ALT, AST, and TBIL levels across different groups of children with community-acquired pneumonia complicated by liver injury. **(A–C)** ALT, AST, and TBIL levels across different age groups of children with community-acquired pneumonia complicated by liver injury ***p < 0.001, ns, no significant difference. **(D–F)** ALT, AST, and TBIL levels in different pathogen infection groups of children with community-acquired pneumonia complicated by liver injury. *p < 0.05, ***p < 0.001, ns, no significant difference. ALT, alanine aminotransferase; AST, aspartate transaminase; TBIL, total bilirubin; MP, *Mycoplasma pneumoniae*.

Among the 1,525 children with CAP complicated by liver injury, 1,403 (92%) had mild elevation of transaminases, 113 (7.41%) had moderate elevation of transaminases, and 9 (0.59%) had severe elevation of transaminases. There were differences in the distribution of liver injury severity within the same age group (*p* < 0.05) (Table 3). The ALT values varied across different age groups. Children in the >1–3-year, >3–5-year, and >5-year groups had higher ALT levels than those in the >28 days to 1-year group [100.5 (78.5, 138) U/L vs. 92 (79.5, 138) U/L vs. 98.5 (81, 139) U/L vs. 86 (76, 109) U/L]. However, there were no significant differences in ALT values among the >1–3-year, >3–5-year, and >5-year groups (Figure 6A). The children were categorized into four groups based on infectious pathogens, with their ALT values showing significant differences. The *M. pneumoniae*-infected group had significantly higher ALT levels than the other three groups. In comparison, the mixed infection group had higher ALT levels than both the *bacterial* infection and *viral* infection groups [99.5 (81, 140) U/L vs. 92.5 (78, 127) U/L vs. 84 (74, 103.5) U/L vs. 87 (76.5, 107) U/L] (Figure 6D). No differences were observed in the aspartate transaminase (AST) and total bilirubin (TBIL) values among children with CAP complicated by liver injury across different age groups or pathogen infection types (Figure 6).

**Table 3:** Degrees of liver injury among children of the same age group.

Age	Mild	Moderate	Severe	χ^2^ value	*p-*value
>28 days–1 years	982 (93.70)	65 (6.20)	1 (0.10)	21.545	<0.001
>1–3 years	178 (85.99)	28 (13.53)	1 (0.48)	13.080	0.001
>3–5 years	84 (94.38)	3 (3.37)	2 (2.25)	6.537	0.038
>5 years	159 (87.85)	17 (9.39)	5 (2.76)	17.896	<0.001

The data presented as *n* (%).

### Characteristics of patients with non-severe or severe community-acquired pneumonia complicated by liver injury

As shown in Table 4, the levels of ALT was higher in children with SCAP compared to those with non-severe community-acquired pneumonia (NSCAP) (*p* = 0.005). The incidence of liver injury is higher in SCAP children (*p* < 0.001). Other clinical and laboratory tests displayed no significant difference.

**Table 4:** Clinical and laboratory characteristics of non-severe and severe community-acquired pneumonia children.

Variable	NSCAP (*n* = 1,214)	SCAP (*n* = 311)	χ^2^ or *Z*-value	*p-*value
Age, months	6 (3, 23)	7 (3, 36)	−1.888	0.059
Male, *n* (%)	713 (58.73)	183 (58.84)	0.001	0.972
Incidence of liver injury, (%)	4.92	8.66	86.059	<0.001
ALT, U/L	87.7 (86.0, 125.0)	103.5 (88.5, 135.0)	−2.786	0.005
AST, U/L	98.0 (81.5, 124.5)	92.0 (76.0, 125.0)	−1.874	0.061
TBil, µmol/L	5.5 (3.7, 9.5)	5.5 (4.0, 8.0)	−0.559	0.576
Degrees of liver injury				
Mild, *n* (%)	1,120 (92.26)	283 (91.00)	0.534	0.465
Moderate, *n* (%)	88 (7.25)	25 (8.04)	0.225	0.635
Severe, *n* (%)	6 (0.49)	3 (0.96)	0.304	0.581^a^

The data presented as M (P25, P75) or *n* (%). NSCAP, non-severe community-acquired pneumonia; SCAP, severe community-acquired pneumonia.

^a^ Continuity Correction.

## Discussion

CAP is an infection affecting the lung parenchyma and/or interstitium and typically presents with fever, cough, and polypnea. Diagnosis is confirmed through pulmonary imageological examination (12). In addition to respiratory manifestations, extrapulmonary complications are not uncommon in children with CAP, with liver injury being one of the recognized complications (6). Liver injury in CAP occurs due to hepatocyte damage and increased membrane permeability, leading to the release of intracellular enzymes into the bloodstream and a subsequent elevation of liver enzyme levels. ALT is predominantly found in hepatocytes and has the highest intracellular concentration among liver enzymes. When hepatocytes undergo degeneration and necrosis, increased membrane permeability results in ALT release into the bloodstream, making it the most direct and sensitive indicator of liver dysfunction (13). Therefore, this study utilizes serum ALT levels as an indicator to assess the severity of liver injury in children with CAP.

In recent years, several studies have explored the etiological and epidemiological characteristics of CAP (14, 15). However, there is limited information regarding the severity of CAP-associated liver injury and pathogen distribution. In this study, we conducted a retrospective analysis of 1,525 children with CAP complicated by liver injury who were hospitalized at the Children's Hospital of Soochow University between January 2018 and December 2022. This study found that the number of CAP children complicated by liver injury decreased from 2020 to 2022 compared with the two prior years (Figure 1). Notably, the number of affected children in 2020 was significantly lower than in the other four years. This may be related to the varying annual incidence rates of children with CAP complicated by liver injury in the Suzhou region. Following the implementation of prevention and control measures in January 2020 due to the *COVID-19* pandemic, there was a significant decline in the mobility and gatherings of people in Suzhou. Additionally, the use of masks in public areas by both travelers and children greatly diminished the risk of cross-infection, contributing to a decline in CAP prevalence. Following the reduction in the prevention and control response level, there was a rise in public gatherings, which led to a corresponding increase in children with CAP. Consequently, the number of children diagnosed with CAP complicated by liver injury increased in 2021 and 2022 (16, 17).

The exact mechanism of CAP-associated liver injury remains unclear. However, it is believed to result from direct pathogen invasion, indirectly by systemic inflammatory responses, and hepatocyte hypoxia, all of which contribute to liver injury (18–20). Among the 1,525 children with CAP complicated by liver injury, the >28 days to 1-year age group had the highest number of cases. Additionally, the incidence of liver injury in this age group was significantly higher than that in the other three age groups. This may be attributed to the relatively low immune function and immature liver development in this age group, making them more susceptible to pathogen-induced liver injury. Therefore, the first year of life represents a critical period for liver function monitoring in children with CAP. Early detection of liver dysfunction allows for timely intervention and symptomatic treatment to prevent further complications.

This study found that the infectious pathogens in children with CAP vary across different age groups. In the >28 days to 1-year group, *viral* infections were the most prevalent, whereas *M. pneumoniae* infections were the least common. As age increased, the proportion of *viral*, and *bacterial* infections gradually decreased while the proportion of *M. pneumoniae* infections increased. These findings align with previous studies on pathogen distribution in children with CAP (21, 22). For CAP caused by *bacterial* infections, *H. influenzae*, and *S. pneumoniae* were the two most common pathogens, while *RSV*, *HRV*, and *HPIV* were the most frequently detected *viruses* in *viral* pneumonia (23, 24). However, the characteristics of pathogen distribution in children with CAP complicated by liver injury in the Suzhou area differed from those of general children with CAP. In the >28 days to 1-year group, there were differences in the constituent ratios of *S. aureus*, *M. catarrhalis*, *M. pneumoniae*, and various mixed infections between the children with CAP and those with CAP complicated by liver injury. In children aged >1–3 years, a significant difference was observed in the constituent ratio of *bacterial*, *viral*, and *M. pneumoniae* mixed infections between children with CAP alone and those with CAP complicated by liver injury. In children aged >5 years, differences were noted in the constituent ratios of *H. influenzae*, *M. pneumoniae*, *FluB*, ≥2 *viral* infections, and *bacteria*-*virus* mixed infections between both groups. These findings indicate that the incidence of liver injury varies by pathogen type and age group. In the >28 days to 1-year group, *bacterial*, and *M. pneumoniae* mixed infections had the highest incidence of liver injury. In the >1–3-year group, *bacterial*, *viral*, and *M. pneumoniae* mixed infections were associated with the highest incidence of liver injury. In the >5-year group, *FluB* infection had the highest incidence of liver injury. Therefore, special attention should be paid to liver function in children with CAP who are infected with the above-mentioned pathogens in each age group.

Among the 1,525 children with CAP complicated by liver injury, 1,403 had mild elevation of transaminases, while only a small number exhibited severe liver injury. The liver injury observed in these children was of the hepatocellular type, with bilirubin levels remaining within the normal range, indicating no impairment in bile excretion. Importantly, none of the children progressed to liver failure. These findings suggest that, in clinical practice, most children with CAP children experience only a mild liver injury. After treatment targeting the primary disease, the children recovered well.

This study demonstrated that ALT values among children with CAP complicated by liver injury varied across different age groups. Children in the >1–3-year, >3–5-year, and >5-year groups had higher ALT levels than those in the >28 days to 1-year group. This study also revealed that ALT values vary depending on the infectious pathogens in children with CAP. Children infected with *M. pneumoniae* had significantly higher ALT levels than those in the other three groups. Additionally, the ALT levels in the mixed infection group were higher than in the *bacterial* and *viral* infection groups. In the >28 days to 1-year group, most children were infected with *viruses* or *bacteria*, which correlated with milder liver injury compared to older children. Among the 1,525 children in this study, mild elevation of transaminases were the most common, with only nine cases of severe liver injury. An analysis of the pathogen characteristics in these nine children indicated that seven cases involved mixed infections, and eight were accompanied by *M. pneumoniae* infection (Table 5). Therefore, greater clinical vigilance is required for CAP accompanied by *M. pneumoniae* infection, as there is a possibility of severe liver injury in these children. Routine liver function tests should be performed to ensure early detection and timely intervention. This study found that children with SCAP are more likely to have elevated transaminases, and the ALT levels in the SCAP group are higher than those in the NSCAP group. Therefore, it is speculated that liver injury may be related to the severity of CAP.

**Table 5:** The pathogen characteristics in 9 children with severe liver injury.

Case	Age	Pathogen
1	>28 days–1 year	RSV + ADV
2	>1–3 years	*M. pneumoniae*
3	>3–5 years	*H. influenzae* + *M. pneumoniae*
4	>3–5 years	*P. aeruginosa* + *M. pneumoniae*
5	>5 years	*S. pneumoniae* + *M. pneumoniae*
6	>5 years	*M. pneumoniae*
7	>5 years	*S. pneumoniae* + *M. pneumoniae*
8	>5 years	*S. pneumoniae* + *M. pneumoniae*
9	>5 years	ADV + HPIV + *M. pneumoniae*

RSV, respiratory syncytial virus; ADV, adenovirus; *M. pneumoniae*, *Mycoplasma pneumoniae*; *H. influenzae*, *Haemophilus influenzae*; *P. aeruginosa*, *Pseudomonas aeruginosa*; *S. pneumoniae*, *Streptococcus pneumoniae*; HPIV, human parainfluenza virus.

This study has some limitations. First, this is a single-center retrospective study, with a relatively small number of children with CAP complicated by liver injury due to certain pathogens. Second, pathogen detection specimens were collected from nasopharyngeal aspirates, which may not fully represent the causative pathogens of pulmonary infections. Third, this study focused on liver injury in children with CAP infected with common pathogens. Future research should involve multi-center, large-sample prospective clinical studies to validate these findings. Additionally, conducting more extensive pathogen screening and monitoring the outcome of liver injury in children with CAP complicated by liver injury can provide more comprehensive clinical guidance.

## Conclusion

Liver injury is a recognized extrapulmonary complication in children with CAP, with mild elevation of transaminases being the most common presentation. The main manifestation was hepatocellular injury rather than cholestatic injury. It occurred most frequently in children with CAP aged >28 days to 1 year. All children basically healed well, none of them progressed to liver failure. Pathogen distribution in CAP complicated by liver injury varied across different age groups. Clinically, greater vigilance is required in *M. pneumoniae* infection, as children with CAP infected by *M. pneumoniae* may be at a higher risk of severe liver injury. Children with SCAP were more prone to liver injury. Although liver injury associated with CAP is often transient and reversible, healthcare workers need to monitor liver function and strengthen supportive treatment. Therefore, in clinical practice, active liver function monitoring of children with CAP should be an essential component of clinical management.

## Data Availability

The raw data supporting the conclusions of this article will be made available by the authors, without undue reservation.
